# *Alternaria* and *Fusarium* Fungi: Differences in Distribution and Spore Deposition in a Topographically Heterogeneous Wheat Field

**DOI:** 10.3390/jof4020063

**Published:** 2018-05-24

**Authors:** Gabriele Schiro, Gernot Verch, Volker Grimm, Marina E. H. Müller

**Affiliations:** 1 Leibniz Centre for Agricultural Landscape Research (ZALF), Eberswalder Str. 84, D-15374 Müncheberg, Germany; verch@zalf.de (G.V.); mmueller@zalf.de (M.E.H.M.); 2 Department Ecological Modelling, Helmholtz Centre for Environmental Research (UFZ), Permoserstr. 15, D-04318 Leipzig, Germany; volker.grimm@ufz.de; 3 Berlin-Brandenburg Institute of Advanced Biodiversity Research (BBIB), Altensteinstr. 34, 14195 Berlin, Germany

**Keywords:** Fusarium head blight, microclimate, canopy, passive spore traps, fungal dispersal

## Abstract

*Fusarium* spp. and *Alternaria* spp., two genera of filamentous fungi, are common colonizers of the wheat phyllosphere. Both can be pathogenic and produce mycotoxins that are harmful to consumers. Their in-field infection dynamics have been a focus for the development of new control strategies. We analysed the abundance on plant ears and spore deposition patterns of *Fusarium* spp. and *Alternaria* spp. in a topographically heterogeneous field. Abundances were assessed genetically, using qPCR-based techniques, and passive spore traps were installed for quantifying the spore deposition at different plant heights. Data loggers were placed to measure the differences in microclimate across the field. Results indicate different distribution and spore deposition patterns for the two fungi. *Fusarium* spp. spore and genetic abundances were higher in spots with a more humid and colder under-canopy microclimate. *Alternaria* spp. showed the opposite trend for genetic abundance, while its spore deposition was not correlated to any of the microclimatic conditions and was more uniform across the field. Our study extends the knowledge on the dispersal and in-field infection dynamics of *Fusarium* spp. and *Alternaria* spp., important for a better understanding of the epidemiology of these wheat pathogens. It also illustrates that topographically heterogeneous fields are a suitable environment for studying the ecology of phyllosphere-colonizing fungi.

## 1. Introduction

Being one of the most important cultivated crops in the world, wheat (*Triticum aestivum* L.) production is threatened by many pests and pathogens that decrease the productivity and quality of the harvested product [1,2]. Particularly destructive is the fungal disease “Fusarium head blight” (FHB), which causes head scab of the wheat plants and hence significant yield losses [3,4]. FHB is caused by a group of 19 species of filamentous fungi, mainly belonging to the genus *Fusarium* (*F.*), with the most important ones in central Europe being *F. graminearum* (teleomorph *Gibberella zeae* (Schwein) Petch) and *F. culmorum* ((W.G. Smith) Sacc (teleomorph unknown)) [3,5]. Most Fusaria produce a multitude of secondary metabolites, considered “mycotoxins” due to their toxic, mutagenic and/or carcinogenic effects on humans and animals once the harvested product is processed into food and consumed [6]. Compounds such as zearalenone (ZEN), deoxynivalenol (DON) or nivalenol (NIV), commonly produced by *F. graminearum* and *F. culmorum*, are considered among the most important classes of mycotoxins due to their acute or chronic toxic effects on mammals [7,8].

Given the economic importance of *Fusarium* spp., much effort went into finding sustainable control methods [9,10]. Ranging from biocontrol strategies to improved managing practices, the development of novel approaches relies on a deep understanding of the ecology of these pathogenic fungi, including their niche preferences, interspecific relationships with other phyllosphere-colonizing microorganisms, and in-field dispersal and infection dynamics [5,11,12]. Focusing on the latter, well known is the important role of debris from previous crops (especially in maize–cereal crop rotations) as an inoculum, where Fusaria can overwinter and sporulate [5,13]. Spores then reach the upper parts of the plant in two different ways: dispersed in the air (wind dispersal) and/or rain-splashed from the ground onto the ear, using plant leaves as intermediate steps [14,15,16]. *Fusarium* species are known to have different spore types. *F. graminearum*, for example, produces ascospores, micro and macronidia, while other species like *F. culmorum* do not produce ascospores because of lacking a sexual stage [17]. Inoculum dispersal is likely to play a major role in determining infections by *Fusarium* species and many studies have investigated the aerobiology of *Fusarium* to clarify its biogeography [18]. Nevertheless, the distribution patterns of spores within the wheat canopy have been rarely studied. Paul et al. [19] observed the rain splash dispersal of spores within the canopy, Manstretta et al. [20] observed differences in deposition patterns between ascospores and conidia of *F. graminearum* in wheat fields. More data and studies are needed to fully understand the in-field epidemiology of FHB. 

Beside the importance of dispersal in disease dynamics, another key factor relevant for the design of control strategies is the relationship of Fusaria with other microorganisms that are commonly found colonizing cereal ears [5]. An important example is the genus *Alternaria* (*A.*) spp. [21]. *Alternaria* are ubiquitous filamentous fungi, described as saprotrophs or opportunistic pathogens, able to colonize a wide range of plants, therefore reported on different types of crops such as small-grain cereals, fruit and vegetables [22]. Species belonging to the genus *Alternaria*, such as *A. alternata* and *A. tenuissima,* have often been associated with cereal diseases like black point, black kernel or leaf blight [23,24,25]. Critical is their ability to produce toxic compounds dangerous for food production, such as alternariol (AOH), alternariol monomethyl ether (AME), altenuen (ALT) and tenuazonic acid (TeA) [22,23,26]. *Alternaria* spp. is known to produce conidia, whose dispersal was studied mostly at the regional level due to the concerns about the allergenicity of inhaled spores. These studies revealed its ubiquitous presence in the air of different cities and environments across Europe [27,28,29]. Both *Fusarium* and *Alternaria* are known to be among the most abundant genera of filamentous fungi colonizing the ear of wheat plants [30,31]. In vitro culture experiments showed that the two fungal genera can influences each other’s growth and metabolic profile, suggesting complex interactions between them during the infection process, which have been described as competitive [32,33]. Field studies on plants observed a negative correlation between their infection rates [34]. Subsequently they have been classified as members of different species clusters, or functional types, which are assumed to have different “lifestyles” or ecological niches [30].

Since both of these genera are key members of the microbial community colonizing wheat ears, the ecological differences and relationships between them deserve special attention, in particular their in-field infection processes. Still, not much is known about the in-field dispersal dynamics of *Alternaria* spp., since, to our knowledge, no studies have so far addressed this question. We used a topographically heterogeneous field as a test plot to observe such differences. Mainly due to heterogeneous topography such as hilltops and depressions and the corresponding differences in water availability, plant productivity differs greatly across the test field. The difference in productivity is reflected in the microclimatic conditions, due to variances in the canopy cover. Well known are the influences of environmental processes on plant infection and colonization, with hyphal growth, spore formation and mycotoxin production being strongly affected by air temperature and humidity [35,36]. Previous studies already showed the within-field differences in abundances of *Fusarium* spp. fungi and their related mycotoxins in heterogeneous fields; specifically, a correlation to soil/air humidity indicators is already known [37]. Since different microclimatic conditions are present but the managing practices and wheat cultivar are the same, heterogeneous fields represent an interesting environment for the study of the phyllosphere community.

We selected a heterogeneous field situated in the Northeast German Lowland, characterised by a high topographic diversity. Microclimate stations were installed in 30 different points across the field according to different water availability. Plant material was sampled at two sampling dates between flowering and harvest. The fungal abundances in the plant material were measured using qPCR-based methods. To study spore deposition patterns, a relatively simple approach, based on the design proposed of Manstretta et al. [20], was used. This method utilizes leaf-like passive spore traps that were placed at different heights at the sampling points mentioned above.

The objective of our study was to detect differences in the abundance of *Fusarium* and *Alternaria* fungi in wheat ears and, simultaneously, their airborne spores across different points of the field, and relate them to microclimatic conditions. We aim at identifying potential ecological differences between these two genera of common wheat phyllosphere colonizers. Moreover, we here provide, for the first time to our knowledge, insights into the dispersal dynamics of *Alternaria* spp. in a cultivated wheat field.

## 2. Materials and Methods

### 2.1. Field Study

**Figure 1:**
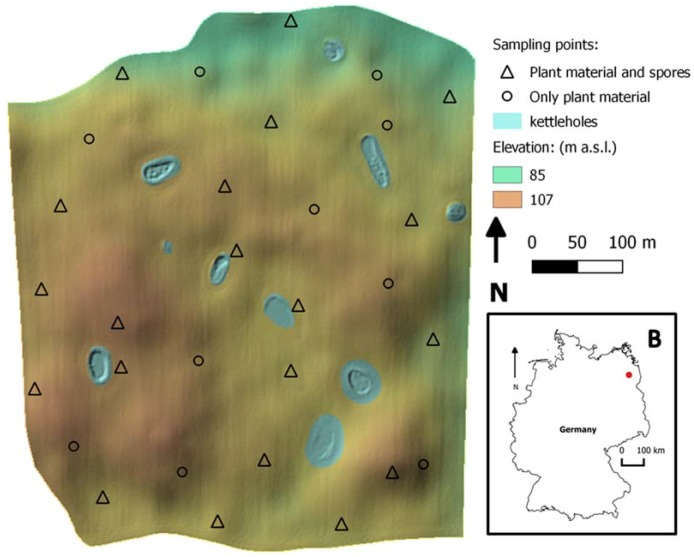
In the main panel, the selected field topography is shown. It is possible to observe its topographic heterogeneity, with hilltops, depressions and small water bodies (kettle holes). With “∆” the location of sampling points with spore traps is shown, and therefore the fungal presence on both plants and spores was quantified. With “O” the location of sampling points without spore traps installed is shown. In panel B, the location of the study field within the German national borders is shown.

The study site is located in the state of Brandenburg (Germany), circa 100 km north of Berlin within the long-term research platform AgroScapeLab Quillow (Agricultural Landscape Laboratory Quillow) of the Leibniz Centre for Agricultural Landscape Research (ZALF) and the Biomove research training group (www.biomove.org/about-biomove/study_area/). The study site is characterized by Pleistocene glacial landforms and sediment, resulting in complex patterns of soils and site conditions with small-scale variations. Due to the glacial deposits, small hills and depressions are typical topographic features that vary over short distances (Figure 1). The field was managed according to standard practices. Wheat plants (cultivar: Julius) grew with maize as the preceding crop after conservation soil tillage (soil cultivation techniques with disc cultivator no more than 0.15 m deep). The susceptibility to *Fusarium* infection of wheat cultivar Julius was 5 (average susceptibility, scales from 1 to 9; [38]).

The field was selected to have a pronounced topographic heterogeneity, and to be subject to crop rotation, specifically wheat after maize to enhance the presence of pathogens [13]. Within the field, 30 points were selected to cover the full range of microclimatic conditions expected due to topographic differences. At each point, a microclimatic station was placed between the wheat canopies. They consisted of a logger “HOBO H21-USB”, which recorded the values measured by air temperature/humidity sensor “S-THB-M002” mounted in a solar radiation shield “RS3”, a soil humidity sensor “S-SMD-M005”, and a leaf wetness sensor “S-LWA-M003”, all of them provided by Onset Computer Corporation (Bourne, MA, USA). Except for the soil humidity sensor, all other sensors were positioned at a height of 30 cm above ground. Sensors provided measurements every hour. In addition, 20 spore samplers were placed at 20 of the points studied across the field.

The experiment started when the wheat plants reached the middle of the milk filling stage (Zadoks scale +/−75) and went on for three consecutive weeks (until the end of the study, grain ripening stage, Zadoks +/−90). During this time the microclimatic stations were active. Spore traps were collected every week. New traps replace the collected ones. Plant height was measured in 15 randomly selected plants in the sampling point at the first sampling date. The average was then used for further analysis. Plant material was collected at each of the 30 points at the beginning and end of the study (first and third sampling date); 15 plant ears were randomly sampled in the square meter surrounding the sampling point. The ears were placed in clean paper bags and transported to the laboratory.

### 2.2. Spore Traps and Samplers

**Figure 2:**
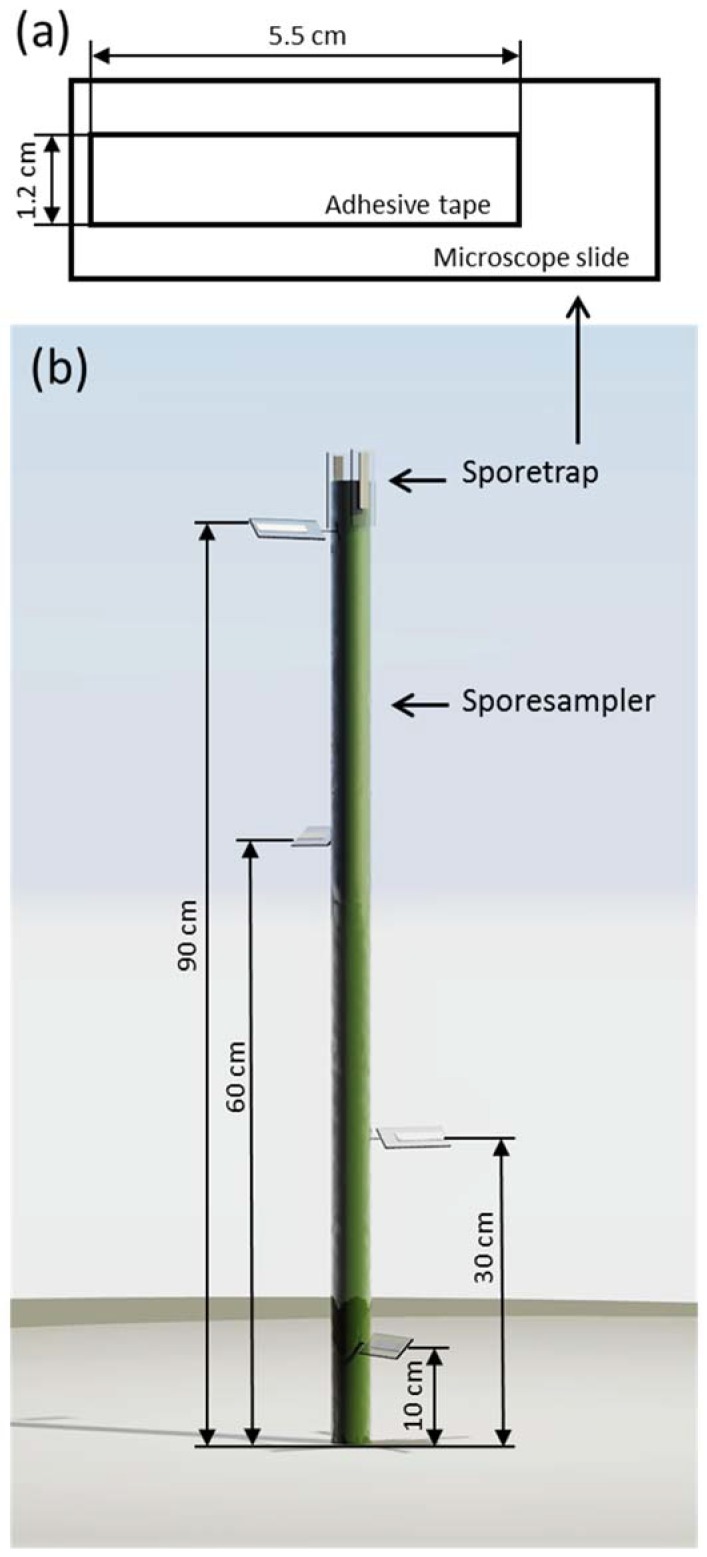
(**a**) The design and dimensions of the spore traps; (**b**) a simplified rendering of the spore sampler, illustrating the placement of the different spore traps.

To observe the spore deposition within the wheat canopy, we used spore traps similar to the design proposed by Manstretta et al. [20]. Spore traps consisted of microscope slides with a piece of transparent double-sided adhesive tape (Article Nr. 64621, Tesa, Beiersdorf, Germany) glued on it. The tape was 1.2 cm wide and 5.5 cm long. These spore traps were placed in the field on a spore sampler (Figure 2). Spore samplers consisted of an aluminium stick, 115 cm long, placed 20 cm into the ground, resulting in an above-ground height of 95 cm. Spore traps were placed at 10, 30, 60 cm and 90 cm above ground. At 10, 30 and 60 cm, one spore trap was placed at different orientations tilted 45° to the ground. At 90 cm, three spore traps were placed, one also tilted 45°, while the other two were parallel to the spore sampler (perpendicular to the ground). One was oriented facing the main wind direction of the area, while the other was faced opposite to the wind direction.

### 2.3. Spore Quantification

Conidia of the genus *Alternaria*, and recognizable spores of the genus *Fusarium*, were counted on each spore trap. In the case of *Alternaria*, spores are easily recognizable due to the “club” shape [21]. We also counted *Ulocladium* spp. spores due to the most recent classification, which includes them in the *Alternaria* genus [39]. In the case of *Fusarium*, only the spores with the typical elongated septated shape are easily recognizable [17]. We therefore excluded micronidia from the counts due to their shape, which make them undistinguishable from other biological materials present in the air under a light microscope. Example photographs of the observed spores are given in the Figure S1. Because of the big differences between the spore traps placed within and outside the canopy, a different counting protocol was used for the two. The lower points showed a higher number of spores, therefore less area was analysed for each spore trap. Spores were counted using a light field standard microscope “Jenaval” (Carl Zeiss, Jena, Germany). At the height of 10, 30 and 60 cm, spores present in an area of 3.14 mm^2^ (which corresponded to the area covered by the field view of the microscope at a magnification of 250×) were counted. Counts were repeated five times at points distributed across the spore trap. For spore traps placed at a height of 90 cm, a total area of 12 mm^2^ was covered. Counts were also in this case repeated five times for each spore trap.

### 2.4. DNA Extraction

The 15 collected ears were dried at 60 °C for 48 h. After drying, they were milled using a laboratory ball-mill MM200 (Retsch, Haan, Germany) at 1000 rpm for 45 s. Once milled, material was carefully mixed and 200 mg was used for DNA extraction using the following protocol. The milled plant material were inserted in a 2-mL centrifuge tube together with 100 µg proteinase K (article nr. 7528.2, Carl Roth, Karlsruhe, Germany) and 1.2 mL CTAB precipitation buffer, composed as follows: 20 g L^−1^ CTAB (article nr. 9161.3, Carl Roth), 1.4 mol L^−1^ NaCl (article nr. 33614, Merck, Darmstadt, Germany), 0.1 mol L^−1^ TRIS (article nr. 37180, Serva, Heidelberg, Germany) and 20 mmol L^−1^ Na_2_EDTA (article nr. 8043, Carl Roth). Samples were then incubated overnight at 65 °C and rotated at 0.5 rotations/s in an incubator “Enviro-genie” (Scientific Industries Inc., Bohemia, NY, USA). Samples were then centrifuged at 10,000× *g* for 10 min. Supernatant was transferred to another tube and 400 µL of chloroform (article nr. 102445, Merck) were added. Samples were mixed by hand for 30 s and then centrifuged at 12,000× *g* for 10 min. Circa 600 µL of superior phase were then transferred to a new clean tube with a double volume of CTAB precipitation buffer. Tubes were then incubated for one hour at room temperature and centrifuged at 12,000× *g* for 10 min. The supernatant was discharged and the pellet resuspended in 350 µL NaCl 1.2 mol L^−1^ solution; 400 µL of chloroform were added and samples were softly mixed by hand for 30 s. The superior liquid phase was transferred to a new tube where 300 µL 4 °C cold isopropanol (article nr. 109634, Merck) were added. Samples were incubated at 4° for 20 min and centrifuged for 15 min at 12,000× *g*. Isopropanol was discharged and pellets were washed with 500 µL 70% ethanol solution (article nr. 111727, Merck). Samples were centrifuged for 15 min at 12,000× *g*. Ethanol was discharged; samples were dried with a “Speedvac DNA 110” (Thermo Fisher Scientific, Waltham, MA, USA). Pellets were dissolved in 100 µL distilled sterile water and stored at −18 °C until further analysis. DNA after extraction reached a concentration between 100 and 100 ng µL^−1^.

### 2.5. Fungal Quantification with qPCR

The total amount of extracted DNA was quantified with a Qubit dsDNA HS Assay Kit (Invitrogen, Waltham, MA, USA). All reactions were carried on a 7500 Fast Real-Time PCR System (Applied Biosystems, Foster City, CA, USA). For *Alternaria*, the genomic DNA of *A. tenuissima* strain name “At 220”, from the microbial collection of the Leibniz Centre for Agricultural Landscape Research (ZALF), was used to produce a standard curve using five consecutive 10 fold dilutions of genomic DNA. DNA was extracted using the protocol from Kahl et al. [40]. The initial concentration of DNA in the standard was calculated measuring the DNA present in the sample and using the genome size for *A. tenuissima* found on the Alternaria genomes database [41]. Self-designed primers and oligoprobes, targeting the alt a 1 gene [42], were used for the estimation of the *Alternaria* spp. abundance. As forward primer “Alt-F1” was used (5′-GCCKGARGGAACCTACTACAACA-3′), as reverse primer “Alt-R1” (5′-GCAGKTGAAGTCGAGDGT-3′) and as locked dual-labelled oligoprobe “Alt-Pr” (5′-FAM-TCAACATCAAGGCYACCAACGGAGG-BHQ-3′). Primer design was based on the sequences published by Hong et al. [42], plus 19 sequences obtained with the same method from samples of Kahl et al. [40] published here (genbank accession number MH375574-MH375592). Measurements were conducted in a 20 µL reaction volume; 0.2 µL samples were added to a solution containing 10 µL TaqMan universal PCR mastermix (Applied Biosystems), 18 pmol of each primers and 5 pmol of fluorescent probe. Reactions were carried out at the following thermal conditions: 95 °C for 10 min and 40 cycles of 95 °C for 15 s and 60 °C for one minute. Primers for *Fusarium* quantification were designed using the genetic region between primers Fa+7 and Ra+6 targeting the translation elongation factor gene *TEF1* [43], using the software “DNA star” (DNASTAR, Inc., Madison, WI, USA). As forward primer “Fa pl3” was used (5′-TACCCCGCCACTCGAGCG-3′), as reverse primer “Fus-pl” (5′-TTGAGCTTGTCAAGAACCCAGGCG-3′) and as locked dual-labelled oligoprobe “Fa pl3” (5′-FAM-CAATAGGAAGCCGCTGAGCTCGGTAAGGGTTC-BHQ-3′). The PCR reactions followed this thermal protocol: 95 °C for 10 min and 40 cycles of 95 °C for 15 s, 65 °C for 20 s and 72 °C for 30 s. The total reaction volume was 20 µL, containing 4 µL of 5× HOT FIREPol^®^ Probe Universal qPCR Mix ROX (Solis Biodyne, Tartu, Estonia), 3 pmol of the corresponding forward and reverse primers, 6 pmol of fluorescent probe and 0.2 µL of template DNA. The genomic DNA of *F. graminearum* strain name “Fg23”, from the microbial collection of ZALF, was used to produce a standard curve with using five consecutive 10 fold dilutions of genomic DNA. Initial copies were calculated using the genomic size presented by King et al. [44].

### 2.6. Statistical Analysis

Statistical analyses were performed using R software version 3.4.2 [45]. Spearman correlation matrixes were created using the package “psych” [46] and a false discovery rate correction, based on the Benjamini Hochberg procedure (*α* = 0.05). To evaluate the effect of wind direction, the spore counts for the two groups (90 cm B, facing main wind direction and 90 cm C, opposite the main wind direction) were compared with a paired *t*-test, where each sampling point was a pair. Averages of all values recorded in the three weeks of the experiment were calculated for each microclimatic parameter at each sampling point. These values were then used for our further analysis. For the relationship between spores and microclimate, repeated measures correlations (as for repeated measurements the three sampling dates), were tested against the microclimate variables using the package “rmcorr” [47]. Spore counts were transformed logarithmically before the test in order to achieve a linear relationship. For both fungal genera abundance measurements and at both sampling dates, multiple regression analysis was used to test if environmental parameters and the genetic abundance of the other genus were significant predictors. Also in this case, qPCR genetic abundance data was previously transformed logarithmically. Beta values (β) were calculated with the package “lm.beta” [48].

## 3. Results

### 3.1. Microclimatic Measurements

**Figure 3:**
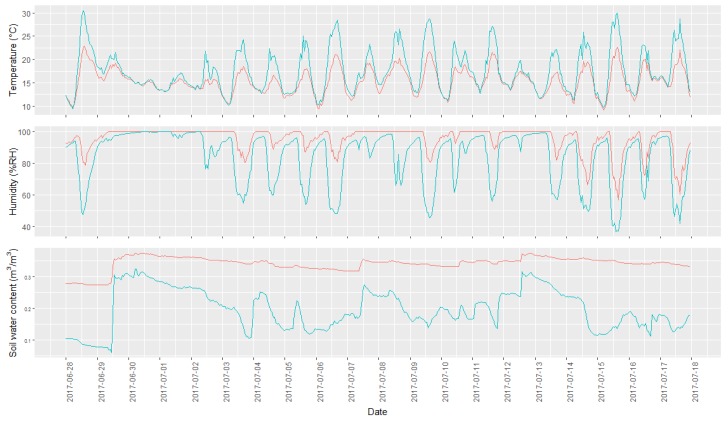
Overview of the range of observed microclimatic data collected during the studies time and with an hourly resolution. The two lines represent the data from the sampling points with the highest and lowest average per microclimatic condition. The green line represents the sampling point with the highest temperature and lowest humidity, while the red line represents the sampling point characterized by a lower air temperature and higher air humidity. In the top graph, the temperature profile is shown, in the middle the humidity profile, and at the bottom the soil water content.

Two of the 30 microclimatic stations did not record any data due to technical failures; these points were therefore discarded from the data analysis. An overview of the measured data is present in Figure 3, where the microclimatic measurements of the point having the maximum and minimum value for each variable measured are represented. The figure shows how different points exhibit very different microclimatic conditions, with daily fluctuations being more pronounced at warmer and less humid points. Soil humidity values showed a wide range of variability, ranging between highly variable and relatively constant values.

A Spearman correlation matrix was created in order to evaluate the relationships between the different microclimatic conditions measured (Table 1). The correlations indicate a strong connection between temperature, humidity and plant height, while the correlations were weaker but still significant for soil humidity with the other variables. This effect is understandable considering the position of the microclimatic stations, situated at a height of 30 cm within the canopy. The measurement is influenced by the canopy shading, which reduces the temperature and raises the humidity. Taller plants are more productive, have more leaves and have a higher canopy cover effect. A higher plant height is therefore positively correlated to humidity and negatively to temperature.

### 3.2. Spore Counts

**Figure 4:**
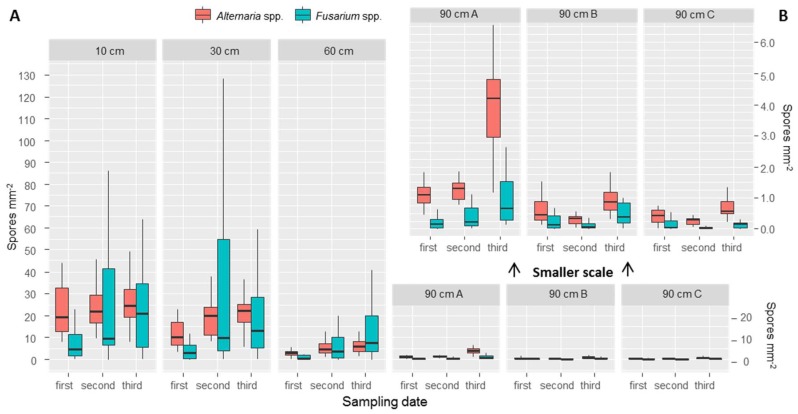
Boxplots representing the values of counted spores per point at different heights sampled at the three different sampling dates. At 90 cm three traps were placed: “90 cm A” were tilted 45 °C, “90 cm B” were against the wind direction (therefore more spores expected), while “90 cm C” were placed hidden from wind. Outliers were omitted due to readability issues. In panel A, a bigger scale is used. In panel B, the same data as 90 cm A, B and C was plotted at a smaller scale, for improving the readability.

We found, on average (with values in brackets indicating the standard deviation between points), 21.5 (30.1) *Fusarium* spores mm^−2^ at the first sampling date, 89.7 (123.0) at the second date and 73.6 (68.4) at the third date. For *Alternaria* we counted 42.7 (25.0) spores mm^−2^ during the first sampling date, 52.1 (19.1) at the second and 31.4 (9.0) at the third. From our results it appears that *Fusarium* spores vary more both across the points and across the three different sampling dates, while *Alternaria* shows more similar results across sampling dates and points. Spore traps placed at different heights allowed us to observe the number of spores deposited at 10, 30 and 60 cm, and outside of the canopy at 90 cm. The results show a decrease in number of spores for both *Fusarium* and *Alternaria* at all three sampling dates (Table 2, Figure 4). While between 10 and 30 cm the differences were small (Figure 4A), a clear difference was observed with 60 cm and with the three top traps placed at 90 cm (Figure 4B).

At 90 cm height, it was possible to observe differences between spore traps tilted by 45° (called “90 cm A”), spore traps placed facing the main wind direction (called “90 cm B”) (therefore expected to receive more spores), and spore traps placed opposite to the main wind direction (called “90 cm C”). A detailed view is presented in Figure 4B. Spore trap “90 cm A” collected more spores; fewer spores were found in the two placed vertically. To evaluate the effect of wind, we compared the group of samples from “90 cm B” and “90 cm C” with a paired *t*-test. Results are presented in Table 3. Although averages of 90 cm B are all higher than “90 cm C”, a *t*-test evidenced statistically significant differences (significance level of *α* = 0.05).

### 3.3. Results of qPCR Measurements

qPCR measurements were taken only at the beginning of the experiment and at the end, therefore we only have the first and the third sampling dates.

**Figure 5:**
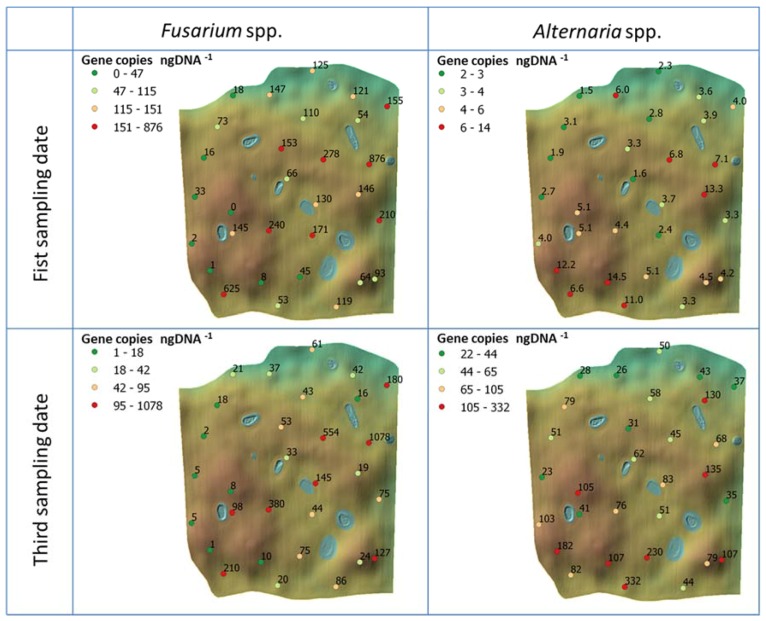
Field maps with specific values of the genetic abundance for both *Fusarium* and *Alternaria* fungi measured at each point in the field at each sampling date (only first and third for the genetic abundance). Colours are based on the gene copies number per nanogram DNA^−1^ and are classified in quartiles.

A spatial map of the obtained infection rates for the two sampling dates is presented in Figure 5. The average infection at the first sampling date for *Fusarium* was 142.65 gene copies ng DNA^−1^, with a standard deviation of 183.1. For *Alternaria,* the amount observed was much lower, averaging 5.09 gene copies ng DNA^−1^ with a standard deviation of 3.4. At the third sampling date *Fusarium* showed a decreased average abundance, with 115.54 gene copies ng DNA^−1^ and a standard deviation of 217.39, while we detected a dramatic increase for *Alternaria,* for which average was 84.1 and there was a standard deviation of 66.46. Also in this case, *Alternaria* showed lower standard deviation values compared to *Fusarium*, meaning a more uniform distribution. A paired *t*-test was performed to check if the differences in abundance between the sampling dates were statistically significant. As pairs, the same sampling locations were used. For *Fusarium* the *t*-test resulted in a *p*-value of 0.21 (therefore not statistically significant at a significance level of 0.05), while for *Alternaria* the *t*-test gave a *p*-value < 0.0001.

### 3.4. Relationship between Microclimatic Conditions and Spore Deposition

**Figure 6:**
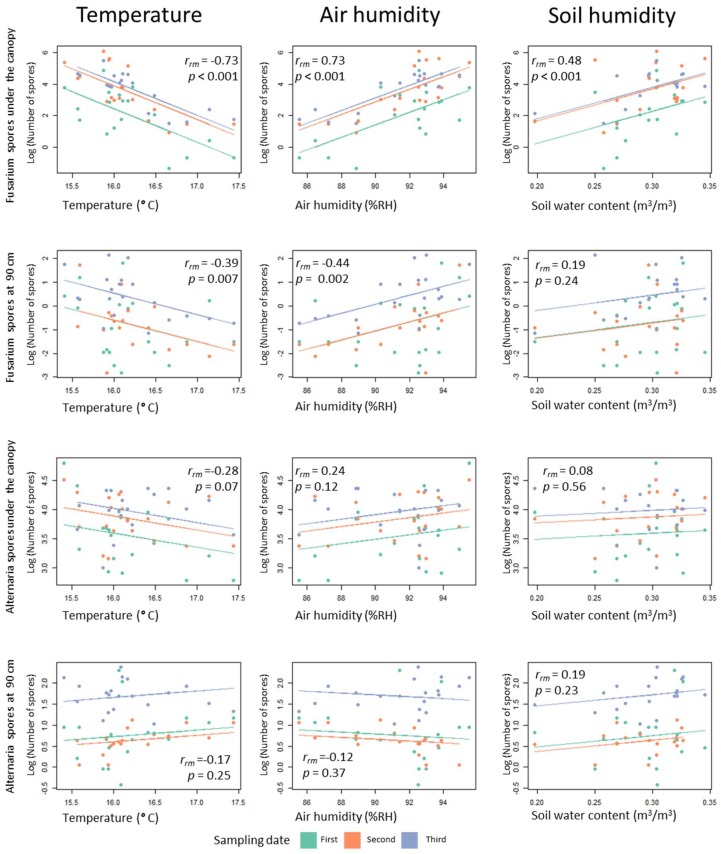
Correlation matrix between environmental parameters and number of spores counted of *Fusarium* and *Alternaria* fungi. The three sampling dates are represented in different colours. The lines show rmcorr fits for each sampling date. These are the best linear fits for each sampling date with the same slope but different intercepts. The rmcorr coefficient (*r_rm_*) shows the strength of the linear association between the two variables. Like a Pearson correlation coefficient, it is a value between +1 and −1. *p*-values are corrected for multiple comparisons using an FDR procedure.

In order to observe possible relationships between the environmental conditions (microclimatic and plant height) and the number of spores counted on the spore traps, a correlation matrix was formed (Figure 6). While *Alternaria* distribution does not show any statistically significant correlation with environmental parameters, *Fusarium* shows significant correlations with temperature, humidity and with soil humidity (the latter for spore traps placed under the canopy). Correlations are stronger for points placed under the canopy.

### 3.5. Relationship between Microclimatic Conditions and qPCR Results

To observe possible relationships between the microclimatic conditions and the genetic abundance at each point detected by qPCR, a Spearman correlation matrix was formed (Table 4).

For multiple regression analysis, only temperature and soil humidity were selected as response variables, together with the abundance of the other opposite genus. Humidity and height were discharged due to the high correlation coefficients of the other variables. Results of the fitted multiple regression analysis show a stronger influence of environmental variables at the first date of sampling for both *Fusarium* spp. and *Alternaria* spp. (Table 5). Besides the opposite behaviour of the two fungi, it is possible to see how soil humidity is a stronger indicator for *Fusarium* compared to *Alternaria*, while temperature is stronger for *Alternaria*. The presence of the other genus could also have a potential effect, since it has shown a nearly significant value in three of the four observations. Noticeable is the disappearing trend at the third sampling date, where patterns are not clearly visible anymore, especially for *Fusarium*, where the model loses its statistical significance (Table 5). For *Alternaria* the disappearing trend is less visible but still present, with the adjusted *r*^2^ dropping to 0.22 but not losing its statistical significance.

## 4. Discussion

For two genera of filamentous fungi common colonizers of the wheat phyllosphere and mycotoxin producers, we explored spore deposition patterns and the abundance of genetic markers across a heterogeneous wheat field and related them to in-field microclimate. We identified different spore deposition and infection abundance patterns for the two genera. In particular, *Fusarium* spores and genetic abundances were higher with a higher humidity and a lower temperature, conditions indicating higher canopy cover and hence productivity. *Alternaria* showed contrasting behaviour; while its genetic abundance is enhanced in drier and warmer spots, the amount of spores caught do not relate to any of the microclimatic variables tested.

We found a clear vertical trend of spore deposition, with more spores for both genera trapped at lower positions. For *Fusarium*, such a vertical trend was also found in earlier studies, with rain-splash dispersal being considered responsible for such patterns [19,20]. Manstretta et al. [20] used a similar method to quantify the spore deposition. The main difference in their findings is in the amount of spores counted; while values seem similar at a height of 90 cm, high differences are found at lower sampled heights. Manstretta et al. [20] reported average values of spores between 20 and 60 spores cm^−2^, while we observed much higher values, on the order of 10 to 100 spores mm^−2^. Since our results underline the connection between spore abundance and canopy-driven microclimate, these differences might be connected to the locations of the spore traps within the field. Manstretta et al. [20] also distinguished between ascospores and conidia, while we did not, since spores were observed to have the same inoculum potential [49].

*Fusarium* spp. spore abundance was correlated with the local environmental parameters measured, such as temperature and plant height at all three sampling dates, also at a height of 90 cm. Previous studies observed spore density in the air in a cultivated field in relationship to weather conditions changing over time; *Fusarium* spores were often found to increase with precipitation and periods of high humidity [50,51]. However, these studies had a different approach: while they studied the aerial spore density over the canopy level, we observed their deposition patterns on the spore traps, which could be seen as “leaves simulators”. Therefore, different factors could explain their positive correlation to the measured microclimatic conditions. Colder and more humid points are characterized by a higher canopy shade. The presence of more leaves and higher plants around spore traps could protect them from the wash-off effect of rain, or simply provide more spore sources, with the leaves acting as a connecting “bridge” for the spore to disperse, as speculated by Xu [11]. Canopy density could also influence the biological activity of fungi in the lower part of the canopy. A higher canopy cover could be responsible for higher water activity in the soil and in crop residues, which was observed to be connected to the microbial activity on substrates [13].

The same trend was observable for *Fusarium* plant infections measured with qPCR, with a higher infection observed in more productive and therefore more humid, less warm sampling points. These results confirm the previous measurements performed by Müller et al. [37], where a correlation between *Fusarium* infection rates and mycotoxins with NDVI as an index of productivity was observed. Current explanations of these trends can only be speculative because the microclimatic conditions were measured under the canopy cover and not at ear height. Caution is needed when drawing conclusions about the ability of the fungus to germinate and grow at different conditions because this would require the measurement of microclimate at ear level. Nevertheless, the connection of the two results, spore deposition patterns and abundance distribution, indicates a strong dependency of *Fusarium* spp. on its short-distance passive dispersal because the number of spores reaching the ears of the plants as inoculum are then dominating the detected local abundance of the fungus in the ear tissue. Still, previous studies argue that the relative role of long- and short-distance dispersal as an inoculum source for infection might differ and depend on the specific weather conditions of a field and its region [52].

For *Alternaria* spp. we were also able to observe a vertical gradient in spore abundances, but microclimatic variables did not have any explanatory power when describing the spore deposition within the field. Still, the local environment seemed to strongly influence the infection abundance on plants, where *Alternaria* is more abundant in dryer and warmer spots. This could be due to the saprophytic nature of *Alternaria*, which grows better when plant tissues are under stress [21]. Previous studies highlighted the importance of certain fungal taxa such as *Alternaria* as both unharmful endophytes and saprobes once the plant grows weaken, losing defence mechanisms [53,54]. To our knowledge, no studies have so far addressed the relationship between water stress and saprophytic fungi present on wheat, and further research would be helpful to fulfil this knowledge gap, also with a view to different climate scenarios of the future. 

Differences between *Fusarium* and *Alternaria* are also observable if we look at the distribution of values both for spore deposition patterns and plant infection results, with *Fusarium* having a coefficient of variation generally higher than *Alternaria* for both spore deposition results and plant infection results, indicating a higher variability across the field, compared to *Alternaria*. Both fungi spore distribution and infection abundance were less correlated with local variables on the second sampling date. This could indicate that, over time, fungi spread and reach plants at sites with less favourable conditions so that infection potentially spreads over the entire field. Also noteworthy is the strong increase in *Alternaria* abundance at the second sampling date. It is known that *Alternaria* becomes more abundant close to harvest or if the harvest is delayed [55]; therefore, the trend could be connected to this phenomenon.

Differences in the dispersal strategies and niche preferences between the two genera of fungi seem clear from our data, with *Fusarium* having pathogenic behaviour and mostly colonizing healthy plants led by a dispersal advantage in more productive spots, while *Alternaria* infection trends seem to be mostly dependent on its host preferences rather than dispersal abilities. These differences could be the reason why these two fungi have been observed as having a negative correlation by Kosiak et al. [34], while it reinforces the results of Nicolaisen et al. [30], which described the two genera as belonging to different clusters based on their lifestyle. Similar results were also obtained by Andersen et al. [56], who showed weather-related differences in infection rates between *Alternaria* and *Fusarium. Alternaria* increased its abundance in dryer years, while *Fusarium* having the opposite trend, causing us to hypothesize an interaction between the two genera happening only on the grain surface. A valuable addition to this study would be to go beyond the genus level, to observe the distribution of single species within the genera *Fusarium* and *Alternaria*, since both genera have shown differences in aggressiveness and ability to colonize different hosts. In the case of *Fusarium*, species such as *F. graminearum* or *F. culmorum* are considered more aggressive than species such as *F. poae* [5]. In the case of *Alternaria*, species belonging to the *A. infectoria* species group are known to be common phyllosphere and endosphere colonizers, not producing any harmful mycotoxins, which are instead made by members of the *A. alternata* species group [57,58]. Other valuable extensions to the study would be the observation of shifts in the whole phyllosphere fungal community and in the metabolome of wheat fungi.

While it is important to study the ecology of phyllosphere fungi of staple crops, our study identified marked differences in distribution and dispersal between these important and potentially harmful wheat phyllosphere colonizers in heterogeneous fields. Our study also demonstrates the potential for using these fields as a tool for studying the ecology of plant-associated microbiomes in agricultural crops. We believe that similar approaches are useful for a more complete picture of the processes leading to plant surface and tissue colonization. A deeper knowledge of such mechanisms is needed for the development of biocontrol, precision and modelling approaches for a more efficient control of plant pathogens.

## Figures and Tables

**Table 1:** Spearman correlation coefficients of measured microclimatic variables. In brackets the *p*-values are reported (Significance codes: *** *p* < 0.001, * *p* < 0.05) after (false discovery rate) FDR correction.

	Humidity	Soil Humidity	Plant Height
Temperature	0.91133 ***	−0.48714 *	−0.89771 ***
Air humidity		0.599343 ***	0.801085 ***
Plant height			0.385648 *

**Table 2:** Average number of spores (AVG), standard deviation (STD) and coefficient of variation (CV) counted at different heights of the spore traps and at different sampling dates.

	***Fusarium*** **spp. (Spores mm^−2^)**								
	**First Sampling Date**			**Second Sampling Date**			**Third Sampling Date**		
height (cm)	AVG	STD	CV	AVG	STD	CV	AVG	STD	CV
10	13.22	28.23	2.13	34.56	57.91	1.68	31.93	45.83	1.44
30	3.55	3.78	1.06	46.29	69.53	1.50	22.31	25.04	1.12
60	3.40	9.14	2.69	8.03	10.49	1.31	17.53	24.16	1.38
90 A	0.81	2.00	2.47	0.64	1.12	1.74	1.46	1.98	1.36
90 B	0.31	0.49	1.59	0.14	0.16	1.16	0.60	0.63	1.05
90 C	0.20	0.29	1.45	0.06	0.10	1.80	0.18	0.21	1.16
	***Alternaria*** **spp. (Spores mm^−2^)**								
	**First Sampling Date**			**Second Sampling Date**			**Third Sampling Date**		
height (cm)	AVG	STD	CV	AVG	STD	CV	AVG	STD	CV
10	22.17	12.06	0.54	23.96	10.46	0.44	25.81	10.72	0.41
30	15.11	15.92	1.05	21.06	11.99	0.57	24.91	12.19	0.50
60	2.75	1.47	0.53	5.13	3.35	0.65	6.13	3.05	0.50
90 A	1.34	1.04	0.78	1.30	0.33	0.25	3.98	1.51	0.38
90 B	0.86	1.27	1.48	0.33	0.20	0.59	1.00	0.52	0.52
90 C	0.46	0.36	0.78	0.31	0.25	0.79	0.77	0.63	0.82

**Table 3:** *p*-values of the paired *t*-test between the number of spores counted on 90 cm B spore traps (against wind) and 90 cm C (hidden from wind). Paired samples were coming from the same sampling point. The three sampling dates were tested separately. In bold are the statistically significant *p*-values (*p* < 0.05).

	First Sampling Date	Second Sampling Date	Third Sampling Date
*Alternaria*	0.08152	0.9409	0.074
*Fusarium*	0.2904	**0.04276**	**0.001962**

**Table 4:** Spearman correlations between measured genetic abundances and environmental parameters (Significance codes: ** *p* < 0.01, * *p* < 0.05, *p* < 0.1, FDR corrected).

Fungus	Sampling Date	Temperature	Humidity	Soil Humidity
*Fusarium*	First	−0.41675 *	0.42266 *	0.579639 **
*Fusarium*	Third	−0.36059	0.345813	0.385878
*Alternaria*	First	0.608374 **	−0.60788 **	−0.35742
*Alternaria*	Third	0.482266 *	−0.50049 *	−0.39409

**Table 5:** Results of the fitted multiple linear models. As response variables, the genetic abundances were taken (transformed logarithmically), explained with microclimatic variables. A multiple linear model was fitted per sampling date, for a total of four models. (Significance codes: *** *p* < 0.001, ** *p* < 0.01, * *p* < 0.05, *p* < 0.1, FDR corrected.)

Response Variable: Gene Copies Number	Sampling Date	Adjusted *r*^2^	β Values (Significance Codes)		
			Temperature	Soil humidity	Other Genus’ Abundance
*Fusarium* spp.	First	0.45 ***	−0.47 *	0.54 **	0.40
	Third	0.16	−0.25	0.38	0.12
*Alternaria* spp.	First	0.5 ***	0.75 ***	−0.21	0.36
	Third	0.22 *	0.42 *	−0.25	0.11

## Supplementary Materials

The following are available online at http://www.mdpi.com/2309-608X/4/2/63/s1.
