# Supplementary material for: Alternaria and Fusarium Fungi: Differences in Distribution and Spore Deposition in a Topographically Heterogeneous Wheat Field
Source: J Fungi (Basel). 2018 May 24;4(2):63. doi: 10.3390/jof4020063 (PMC6023320; doi:10.3390/jof4020063)
Supplement: Supplementary file 1 [file jof-04-00063-s001.zip › jof-295304-SI.pdf]

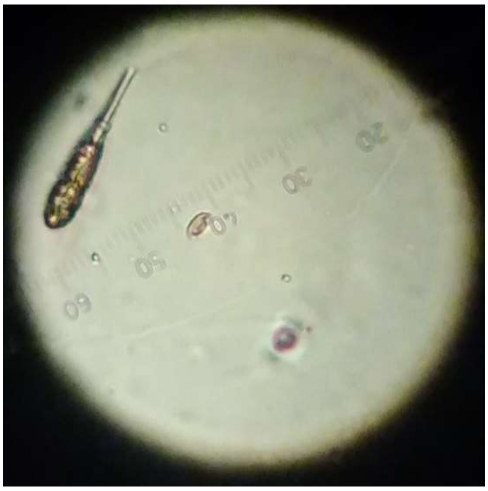

(**a**)

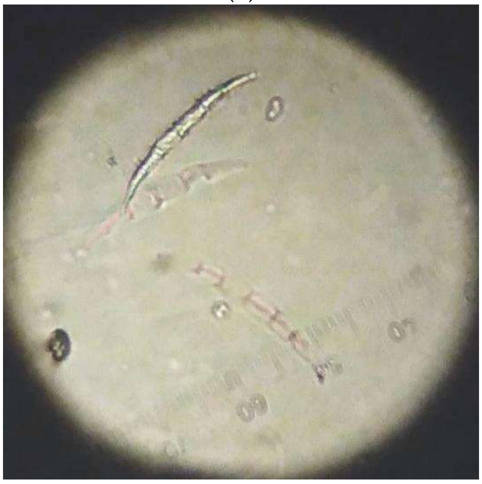

(**b**)

Figure S1. *Alternaria* spore (**a**) and *Fusarium* pores (**b**) observed and counted on the microscope slides.
